# The clinical outcome of implantable collamer lens in corneal ectasia post astigmatic keratotomy

**DOI:** 10.1016/j.ijscr.2025.110855

**Published:** 2025-01-07

**Authors:** Hussam Al-Razqan, Mohammed Al-Mutlak

**Affiliations:** King Khaled Eye Specialist Hospital, Riyadh, Kingdom of Saudi Arabia

**Keywords:** Posterior chamber phakic intraocular lens, Implantable collamer lens, Keratoconus, Corneal ectasia, Astigmatic keratotomy, Myopia, Myopic astigmatism

## Abstract

**Introduction:**

Implantable collamer lens (ICL) is a posterior chamber phakic intraocular lens. It is usually indicated for high refractive error correction that cannot be treated by laser vision correction due to physical limitations.

**Case presentation:**

39 years old male with a past ocular history of keratoconus underwent astigmatic keratotomy followed by crosslinking in both eyes a couple of years later due to signs of corneal ectasia progression.

**Discussion:**

Astigmatic keratotomy is highly unpredictable in keratoconus patients as it might induce progressive irregular astigmatism. After confirming the stability of refraction and corneal topographic reading for the past couple of years. We insert EVO visian V4c ICL 13.2 manufactured by STAAR surgical company under topical anesthesia sequentially to both eyes.

**Conclusion:**

Our case depicts the successful use of ICL in ectatic cornea post-astigmatic keratotomy with an excellent outcome. Further study with large case series and long follow-up is recommended to evaluate the efficacy and safety profile.

## Highlights

• a unique indication of the use of implantable collamer lens (ICL).
• we showed an interesting finding of corneal ectasia progression after astigmatic keratotomy in keratoconus patient.
• Despite the highly unpredictable corneal irregularity after astigmatic keratotomy in keratoconus and the high order aberration induced.
• The ICL excel to show us an excellent outcome of an unusual indication.

## 1 Introduction

Keratoconus is a condition that makes the cornea resemble a conical shape due to noninflammatory steepening of the anterior and posterior curvature with progressive thinning. [1] Astigmatic keratotomy is an arcuate mid-peripheral corneal incision that can be performed with a femtosecond laser. It usually reduces the amount of astigmatism by flattening the cornea in the incision meridian and steepening 90 degrees away. [2] Implantable collamer lens (ICL) is a posterior chamber phakic intraocular lens. It is usually indicated for high refractive error correction that cannot be treated by laser vision correction due to physical limitations. FDA approved the Visian ICL, manufactured by STAAR surgical company since 2005 and has been commercially available in 2011. Since then, several studies have looked at the effectiveness of ICL by evaluating multiple variables that include post-operative uncorrected visual acuity, predictability of refraction, and stability along with its safety profile. [[3], [4], [5], [6], [7], [8]] In this case report, we report the outcome of an unusual indication for an ICL in a patient with corneal ectasia and high myopic astigmatism post astigmatic keratotomy. This case report has been reported in accordance with the SCARE criteria. [9]

## 2 Case report

**Fig. 1:**
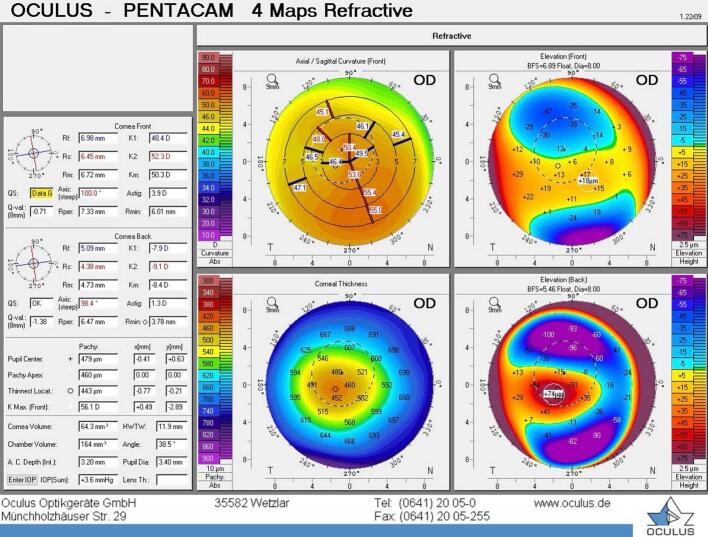
Topographic image of the right eye showing four maps using oculus pentacam device.

**Fig. 2:**
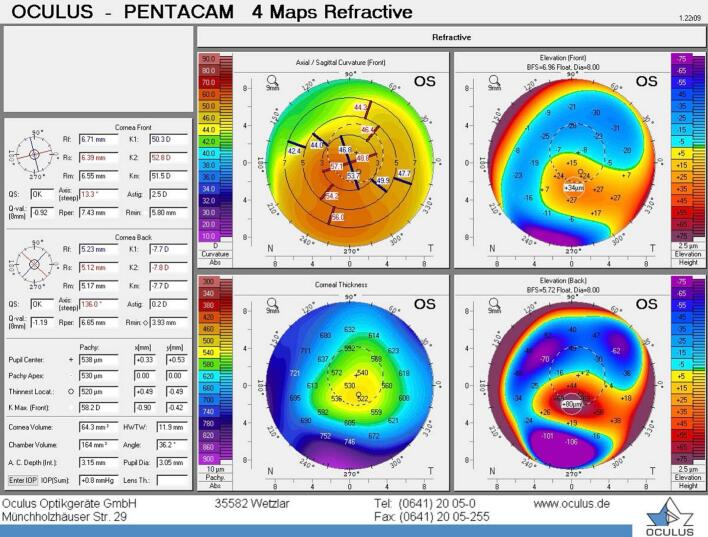
Topographic image of the left eye showing four maps using oculus pentacam device.

A 39-year-old male presented to our facility complaining of poor uncorrected vision in both eyes. His past ocular history is known as keratoconus and had a couple of interventions elsewhere before he presented to us. Those procedures he had elsewhere are astigmatic keratotomy in 2009 for both eyes followed by crosslinking (CXL) in 2013 for the right eye and in 2016 for the left eye. The patient otherwise is healthy and did not have any medical disease. Upon presentation, he had uncorrected distance visual acuity (UCVA) of 0.9 and 1.2 LogMAR to the right and left eye, respectively. His refraction showed high compound myopic astigmatism with the best spectacle-corrected visual acuity (BSCVA) of 0.1 LogMAR in both eyes. His refraction measurement was minus 10 spherical diopters, minus 3 cylindrical diopters at axis of 25 degree, and minus 11.5 spherical diopters, minus 3.5 cylindrical diopters at axis of 95 degree to the right and left eye, respectively. His intraocular pressure was within the normal range in both eyes. A slit lamp examination of the anterior segment showed a clear cornea apart from the previous arcuate astigmatic keratotomy scar in the mid periphery, the lens is clear and posterior segment were unremarkable. His topography reading using oculus pentacam device was as follows for the right and left eye respectively. K1 48.4, k2 52.3, kmax 56.1, corneal thickness (CT) 443 μm, anterior chamber depth (ACD) 3.20 mm, angle 38.5, and white to white (WTW)11.9 mm. (Fig. 1) K1 50.3, k2 52.8, kmax 58.2, CT 520 μm, ACD 3.15 mm, angle 36.2, and WTW 11.9. (Fig. 2).

After confirming the stability of refraction and corneal topographic reading for the past couple of years. We counseled the patient regarding the option of ICL insertion including benefits, risks, and possible complications. Then we order the lens based on the company nomogram. EVO Visian V4c ICL 13.2 manufactured by STAAR surgical company arrived and we inserted it under topical anesthesia sequentially to both eyes. Both surgeries went uneventfully without complications. Using approximately 3.5 mm temporal main wound a toric ICL inserted at 5- and 11-degree axis in the right and left eye, respectively. Routine post-operative medications were given that include topical antibiotic for one week, tapering topical steroid for four weeks, and 500 mg oral acetazolamide for one day. We followed up the patient on day 1 postoperatively, 2 weeks, 8 weeks, and 6 months later. On day 1, UCVA were 0 LogMAR for the right eye and 0.2 LogMAR for the left eye. The patient maintained this vision for more than 6 months post-operatively with no residual refraction. Intraocular pressure (IOP) was within normal range for both eyes. He did not experience a spike of high IOP or angle closure glaucoma during the follow-ups. ICL vault measurement was done using anterior segment optical coherence tomography (AS-OCT). It showed 301 μm for the right eye and 498 μm for the left eye. Upon the last visit, the patient was satisfied and maintained his UCVA, normal intraocular pressure and anterior segment slit lamp examination was unremarkable. Optical phenomena that include halo, glare, and starburst were noticed especially at night. However, it did not limit patient activity.

## 3 Discussion

In our case report, we noticed that the patient developed ectatic corneal progression years post astigmatic keratotomy in which he had to do CXL in both eyes later for stabilization. The previous procedures were performed elsewhere before he presents to our facility. The gap between the two procedures is mostly due to missing follow-up. We attribute this progression due to the biomechanical changes that lead to a further weakening of the cornea post-astigmatic keratotomy. In addition to the course of keratoconus disease. Astigmatic keratotomy is highly unpredictable in keratoconus patients as it might induce progressive irregular astigmatism. Interestingly, our patient still maintained good vision with spectacles. That was encouraging for us to discuss the option of ICL implantation with our patient after confirming the stabilization of his cornea for more than one-year post-CXL. The use of ICL insertion in keratoconus has been performed off-labeled with promising outcomes in the literature. [[3], [4], [5], [6], [7], [8]] Antonios et al. evaluated the ICL implantation for keratoconus patients after six months post-CXL. He showed a significant improvement in visual acuity and refraction in keratoconus patients with high myopia and astigmatism. [10] In a more recent paper, Fairaq et al., demonstrate that ICL implantation can be a safe and effective option in mild, moderate, and advanced keratoconus patients. [11] Our case comes in consistent with the literature regarding ICL implantation in keratoconus. To our knowledge, we did not come across a reported case of ICL implantation in post-astigmatic keratotomy that led to ectasia progression. Our patient post-ICL implantation had UDVA of 0 LogMAR and 0.2 LogMAR in the right and left eye, respectively. He maintained this vision for more than six months post-operatively with no residual refraction that can be corrected with spectacles. ICL vault was within the normal range in both eyes. We did not encounter an incident of high intraocular pressure, pupillary block, iritis, or other post-operative complication. Our case depicts a good predictability of refraction, sizing, and maintaining ICL stability in keratoconus eye post astigmatic keratotomy. Optical phenomena are still believed to be there however the highly refractive error corrected with ICL is the key to patient satisfaction in our case.

## 4 Conclusion

ICL is an effective and safe option in certain cases. Our study demonstrates that ICL is an excellent choice for patients whom excimer laser is not an option and ICL continue to have more unusual indication such as our patient who had ectasia progression and astigmatic keratotomy wound and was managed with an excellent outcome. Further study with large case series and long follow-up is recommended to evaluate the efficacy and safety profile of such a procedure with a certain condition.

## Authorship

All authors attest that they meet the current ICMJE criteria for Authorship.

## Patient consent

Written informed consent was obtained from the patient for publication of this case report and accompanying images. A copy of the written consent is available for review by the Editor-in-Chief of this journal on request.

## Ethical approval

Ethical approval for this study (RD/26001/IRB/0319-23) was approved by the IRB department at King Khaled Eye Specialist Hospital, Riyadh, Saudi Arabia on 09/07/2023.

## Guarantor

King Khaled Eye Specialist Hospital, Riyadh, Saudi Arabia.

## Funding

No funding or grant support.

## Declaration of competing interest

The authors declare that they have no financial interest or personal relationship that could have appeared to influence the work reported in this paper.
